# Role of Endocannabinoids and Cannabinoid-1 Receptors in Cerebrocortical Blood Flow Regulation

**DOI:** 10.1371/journal.pone.0053390

**Published:** 2013-01-04

**Authors:** András Iring, Éva Ruisanchez, Miriam Leszl-Ishiguro, Béla Horváth, Rita Benkő, Zsombor Lacza, Zoltán Járai, Péter Sándor, Vincenzo Di Marzo, Pál Pacher, Zoltán Benyó

**Affiliations:** 1 Institute of Human Physiology and Clinical Experimental Research, Semmelweis University, Budapest, Hungary; 2 Pathology and Laboratory Medicine Institute, Cleveland Clinic, Cleveland, Ohio, United States of America; 3 Department of Cardiology, St. Imre Teaching Hospital, Budapest, Hungary; 4 Endocannabinoid Research Group, Institute of Biomolecular Chemistry, Consiglio Nazionale delle Ricerche, Naples, Italy; 5 National Institute on Alcohol Abuse and Alcoholism, National Institutes of Health, Bethesda, Maryland, United States of America; Massachusetts General Hospital/Harvard Medical School, United States of America

## Abstract

**Background:**

Endocannabinoids are among the most intensively studied lipid mediators of cardiovascular functions. In the present study the effects of decreased and increased activity of the endocannabinoid system (achieved by cannabinoid-1 (CB1) receptor blockade and inhibition of cannabinoid reuptake, respectively) on the systemic and cerebral circulation were analyzed under steady-state physiological conditions and during hypoxia and hypercapnia (H/H).

**Methodology/Principal Findings:**

In anesthetized spontaneously ventilating rats the CB1-receptor antagonist/inverse agonist AM-251 (10 mg/kg, i.v.) failed to influence blood pressure (BP), cerebrocortical blood flow (CoBF, measured by laser-Doppler flowmetry) or arterial blood gas levels. In contrast, the putative cannabinoid reuptake inhibitor AM-404 (10 mg/kg, i.v.) induced triphasic responses, some of which could be blocked by AM-251. Hypertension during *phase I* was resistant to AM-251, whereas the concomitant CoBF-increase was attenuated. In contrast, hypotension during *phase III* was sensitive to AM-251, whereas the concomitant CoBF-decrease was not. Therefore, CoBF autoregulation appeared to shift towards higher BP levels after CB1-blockade. During *phase II* H/H developed due to respiratory depression, which could be inhibited by AM-251. Interestingly, however, the concomitant rise in CoBF remained unchanged after AM-251, indicating that CB1-blockade potentially enhanced the reactivity of the CoBF to H/H. In accordance with this hypothesis, AM-251 induced a significant enhancement of the CoBF responses during controlled stepwise H/H.

**Conclusion/Significance:**

Under resting physiological conditions CB1-receptor mediated mechanisms appear to have limited influence on systemic or cerebral circulation. Enhancement of endocannabinoid levels, however, induces transient CB1-independent hypertension and sustained CB1-mediated hypotension. Furthermore, enhanced endocannabinoid activity results in respiratory depression in a CB1-dependent manner. Finally, our data indicate for the first time the involvement of the endocannabinoid system and CB1-receptors in the regulation of the cerebral circulation during H/H and also raise the possibility of their contribution to the autoregulation of CoBF.

## Introduction

Endocannabinoids (ECs) are endogenous bioactive lipid mediators exerting many of their effects in mammals through their specific G protein-coupled receptors [1]. The main ECs are anandamide and 2-arachidonoyl glycerol (2-AG), the former favoring cannabinoid receptor 1 (CB1) and the latter, cannabinoid receptor 2 (CB2) [2]. These receptors are also involved in mediating the effect of several constituent compounds of the plant Cannabis sativa (marijuana), such as Δ^9^-Tetrahydrocannabinol (Δ^9^-THC) and Δ^9^-Tetrahydrocannabivarin (Δ^9^-THCV) [3]. ECs have been implicated in many physiological functions and also in pathophysiological processes [4], such as diseases and aging of the cardiovascular system [5], [6], ischemia-reperfusion injury [7], hypertension [8], diabetes [9] and obesity [10]. Selective targeting of the cannabinoid receptors [11] or the metabolizing enzymes [12]–[14] is being developed and gives a promising opportunity for therapeutic interventions in the near future.

The cerebral circulation is tightly regulated by neuronal [15]–[17] and humoral mechanisms with the involvement of several major vasoactive factors, such as nitric oxide [18], prostanoids [19]–[21], opioids [22] and carbon monoxide [23]–[25]. The role of the EC system in the regulation of cerebral blood flow (CBF) is, however, still largely unknown. It was recognized very early that Δ^9^-THC is able to increase CBF in dogs [26], and this observation has recently been verified with positron emission tomography in humans [27], [28]. Furthermore, administration of anandamide dilated cerebral arterioles of rabbits [29] and isolated cerebral arteries of cats [30], but caused a decrease in CBF in rats [31]. In other *in vivo* observations in rats, however, both anandamide and the CB1-receptor agonist HU-210 elicited marked cerebral vasodilation, which was inhibited by a CB1-antagonist [32].

To address these contradictory findings and to clarify the role of ECs and CB1-receptors in cerebral circulation, we carried out experiments in rats with the administration of a CB1 receptor antagonist/inverse agonist (AM-251) and an EC reuptake inhibitor (AM-404) under resting physiological conditions and we also examined the role of CB1-receptors in hypoxia and hypercapnia (H/H). In comparison to previous studies, in which the effects of exogenously applied cannabinoids were determined, we aimed to investigate the influence of endogenous cannabinoids by either suppressing or enhancing the activity of the EC system with AM-251 and AM-404, respectively. We show for the first time that while constitutive CB1 receptor activation appears to play a limited role in the maintenance of the resting cerebrocortical blood flow (CoBF), ECs modulate CoBF during H/H in a CB1-dependent manner, an interaction that may have a very important role in pathophysiological conditions associated with altered EC system.

## Materials and Methods

Experiments were performed on adult male Wistar rats (300–400 g) according to the guidelines of the Hungarian Law of Animal Protection (243/1988), and all procedures were approved by the Semmelweis University Committee on the Ethical Use of Experimental Animals (590/99 Rh). Rats were anaesthetized with intraperitoneally (i.p.) applied urethane (1.3 g kg^−1^), the depth of anesthesia was regularly controlled during the experiments by checking the plantar nociception reflex and additional urethane was administered intravenously (i.v.) as necessary. In previous studies we found that this anesthetic regimen provides stability of systemic and cerebrocortical circulatory parameters, arterial blood gas tensions and pH over an experimental period of up to 2.5 hours [33], and others have shown that some cardiovascular actions of intravenous anandamide administration can be observed in urethane but not in pentobarbitone anesthesia [34]. Furthermore, in contrast to volatile anesthetics, such as halothane and isoflurane [35], urethane has not been reported to directly influence the CoBF in rats. The animals were spontaneously breathing through an intra-tracheal cannula. Catheters were inserted into both femoral arteries (for systemic arterial blood pressure measurement and for blood sampling) and into the left femoral vein (for drug administration). Body temperature was kept between 37 and 38°C during the experiments using a heating pad controlled by a rectal probe.

Measurement of the CoBF was performed by laser-Doppler (LD) flowmetry as described in detail elsewhere [33]. Briefly, the head of the animal was fixed in a stereotaxic head holder with the nose 5 mm down from the interaural line. The skull of the parietal region was exposed and the bone was thinned over the parietal cortex on both sides with a microdrill, so that the lamina interna of the skull remained intact. Two LD probes were placed above the thinned skull at a 12°-angle to the vertical to provide an optimal view of the cortex (4 mm caudal from bregma, 5 mm lateral from midline). LD flux (LDF) was measured with a two-channel blood flow monitor (MBF3D, Moor Instruments, UK) and was recorded continuously. The LD monitor was calibrated before each individual experiment with a constant movement latex emulsion. The laser light was in the infrared range (780 nm) and penetrated about 1 mm into the brain covering approximately 7 mm^2^ of the parietal region, so that the data acquired mostly represented the characteristics of the blood flow in the parietal cortex [24]. Blood pressure (BP) and CoBF were recorded continuously (BIOPAC Systems Inc, Goleta, CA, USA); the heart rate was calculated from the pulsating BP signal. Arterial blood gas and pH measurements were performed throughout the experiments by a Radiometer (Bronshoj, Denmark) ABL-77 analyzer and by the use of a capnograph (Capstar-100, CWE Inc., Ardmore, PA, USA). However, if the onset of capnography resulted in a more than 10 mmHg reduction of the arterial O_2_ tension, the device was disconnected and not used in that experiment.

Each animal was tested by one of the following experimental protocols. With the *first protocol* the influence of CB1-receptors on the CoBF under resting conditions was studied. After a 15-min baseline period one experimental group received 1 ml vehicle (containing ethanol/emulphor/saline; 1∶1:8; v:v:v); the other was treated with AM-251 (10 mg kg^−1^ i.v.). Blood samples were taken before as well as 1, 2, 4, 8, 16, and 32 minutes after the administration of AM-251 or its vehicle. The *second protocol* was designed to study the effects of enhanced EC levels on the systemic and cerebrocortical circulation. Following baseline measurements, the animals received a single dose of 10 mg kg^−1^ AM-404 i.v. (dissolved in the same vehicle as AM-251) in order to inhibit the reuptake of ECs. After blood pressure, CoBF, blood gas and acid-base parameters returned to their baseline levels, the animals were randomly divided into two experimental groups receiving intravenously either vehicle or 10 mg kg^−1 ^AM-251. Fifteen minutes later the administration of 10 mg kg^−1^ AM-404 was repeated and the measurements were continued for an additional 45 min. With the *third protocol* the role of CB1-receptors was studied during controlled H/H, which was induced in a stepwise manner by the administration of different gas mixtures (10% O_2_–10% CO_2_–80% N_2_ for producing mild H/H, 5% O_2_–20% CO_2_–75% N_2_ for producing moderate H/H and 20% CO_2_–80% N_2_ for producing severe H/H) with a constant flow of 3 l min^−1^ through a 5-ml open chamber connected to the trachea, at atmospheric pressure. CoBF was recorded continuously and its peak values were determined during the 8-min long steps of H/H. After the first mild, moderate and severe H/H challenge, the animals were randomly divided into two experimental groups receiving either vehicle or AM-251 (10 mg kg^−1^ i.v.). Thirty minutes later the three steps of H/H were repeated in both groups and peak values were determined from the continuous recording of CoBF.

AM-251 (1-(2, 4-dichlorophenyl)-5-(4-iodophenyl)-4-methyl-N-1-piperidinyl-1H-pyrazole-3-carboxamide) and AM-404 (N-(4-hydroxyphenyl)-5Z, 8Z, 11Z, 14Z-eicosatetrenamide) were obtained from Cayman Chemicals (Ann Arbor, MI, USA) and dissolved in 1 ml of vehicle containing ethanol/emulphor/saline (1∶1:8; v:v:v). All other drugs were from Sigma (St. Louis, MO, USA). Values are presented as mean ± SEM; *n* represents the number of experiments. Statistical analysis was performed using ANOVA followed by a Tukey post-hoc test or Student’s paired *t*-test when comparing two variables. A *P* value of less than 0.05 was considered to be statistically significant.

## Results

### Effects of Constitutive Endocannabinoid Release and CB1-receptor activity on Systemic Blood Pressure and Cerebrocortical Blood Flow

**Figure 1:**
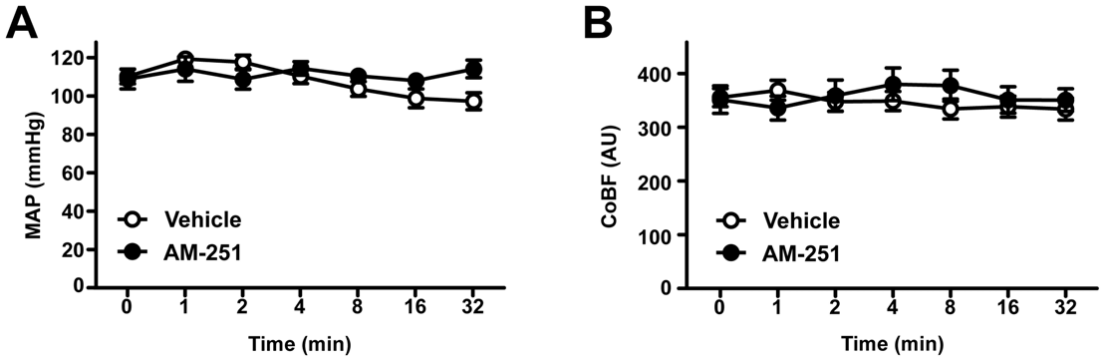
The effects of CB1-receptor blockade on mean arterial pressure (A) and cerebrocortical blood flow (B). Mean arterial pressure (MAP, n = 10) and cerebrocortical blood flow (CoBF, n = 20) are shown before (0 min) as well as 1, 2, 4, 8, 16 and 32 minutes after intravenous administration of 10 mg kg^−1^ AM-251 (•) or its vehicle (○) in urethane-anaesthetized rats. Values are presented as mean ± SEM. No significant difference was found with repeated measures analysis of variance and Tukey’s post hoc test. AU, arbitrary unit.

First, the potential influence of tonic EC release and constitutive CB1-receptor activity on the resting BP and CoBF was studied by i.v. administration of the selective CB1 antagonist/inverse agonist AM-251 in a dose of 10 mg kg^−1^, which had been shown to be effective *in vivo* in previous studies [36], [37]. Vehicle-treated animals served as controls. Neither AM-251 nor its vehicle induced any significant changes in the mean arterial blood pressure (MAP, Figure 1A) or CoBF (Figure 1B) up to 32 minutes after their administration. Furthermore, heart rate, arterial blood gas tensions, acid-base parameters and hematocrit remained unchanged during the observation period (Table 1). These findings indicated that constitutive CB1-activity has probably no significant influence on the systemic and cerebrocortical circulation under steady-state resting conditions in healthy normotensive rats.

**Table 1:** Physiological parameters before (0 min) and at several timepoints after the administration of AM-251 or its vehicle.

	Treatment	0 min	1 min	2 min	4 min	8 min	16 min	32 min
**Heart Rate (bpm)**	*Vehicle*	420±10	402±7	404±7	404±8	413±10	423±10	396±16
	*AM-251*	411±11	384±10	379±12	377±10	385±10	404±9	407±12
**pH**	*Vehicle*	7.40±0.01	7.37±0.01	7.37±0.01	7.38±0.01	7.39±0.01	7.40±0.01	7.41±0.01
	*AM-251*	7.40±0.01	7.40±0.01	7.41±0.01	7.42±0.02	7.42±0.02	7.40±0.01	7.41±0.01
**PaO_2_ (mmHg)**	*Vehicle*	87.7±2.0	76.3±3.2	76.5±2.7	79.0±2.7	78.4±2.7	81.3±3.2	80.2±3.2
	*AM-251*	83.6±2.0	82.3±6.5	85.8±2.1	86.9±1.8	85.4±2.7	84.4±2.0	84.4±2.0
**PaCO_2_ (mmHg)**	*Vehicle*	42.7±1.0	43.5±1.0	43.0±0.9	41.8±1.2	40.4±1.1	39.6±1.3	38.1±1.7
	*AM-251*	40.4±1.5	40.2±1.2	37.3±1.7	36.8±2.2	35.7±1.6	37.4±1.2	38.7±1.4
**O_2_ Saturation (%)**	*Vehicle*	96.5±0.2	93.8±0.9	94.2±0.7	94.8±0.5	95.0±0.6	95.5±0.6	95.6±0.6
	*AM-251*	96.2±0.3	95.2±0.7	96.5±0.3	96.7±0.3	96.5±0.4	96.3±0.3	96.3±0.4
**SBE (mmol/l)**	*Vehicle*	1.2±0.4	0.1±0.4	-0.3±0.4	-0.6±0.4	-0.2±0.5	-0.1±0.5	-0.5±0.6
	*AM-251*	0.1±0.7	-0.6±0.5	-0.9±0.5	-0.8±0.4	-1.3±0.3	-1.3±0.4	-1.2±0.3
**Hematocrit (%)**	*Vehicle*	45.8±1.1	44.1±1.1	43.4±0.9	43.4±1.2	42.9±0.8	43.5±1.2	42.1±1.0
	*AM-251*	42.8±1.3	40.5±1.0	41.0±0.8	42.6±1.2	42.9±0.9	41.9±1.3	41.1±1.9

SBE = Standard Base Excess.

Values are means ± SEM, n = 22 in the vehicle-treated and n = 18 in the AM-251-treated group.

### Effects of Enhanced Endocannabinoid Release and Consequent Activation of CB1-receptors on the Systemic Blood Pressure and Cerebrocortical Blood Flow

**Figure 2:**
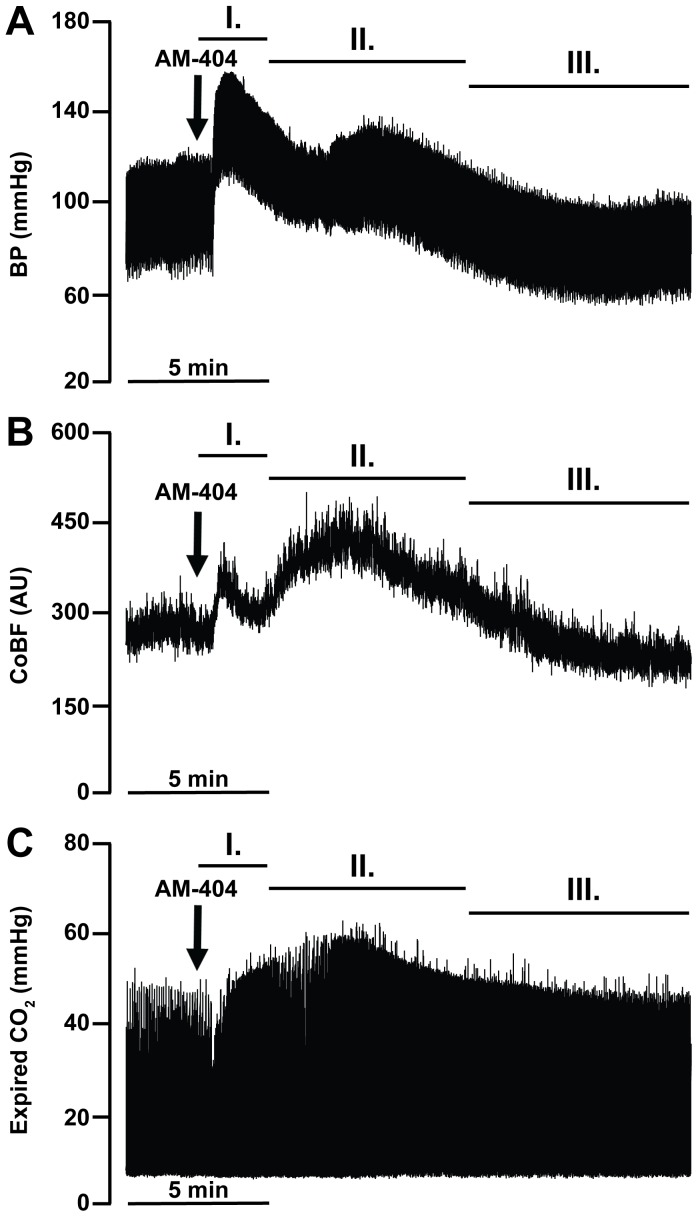
Representative recordings of the effects of endocannabinoid reuptake inhibition on blood pressure (A), cerebrocortical blood flow (B) and expired CO_2_ levels (C). After the intravenous administration of 10 mg kg^−1^ AM-404 a transient increase in blood pressure (BP) and cerebrocortical blood flow (CoBF) can be seen (phase I). It is followed by a second prominent increase in CoBF (phase II) that is accompanied with a rise of expired CO_2_. Thereafter a sustained decrease in the BP and CoBF can be seen (phase III). The arrows indicate the injection of AM-404.

**Figure 3:**
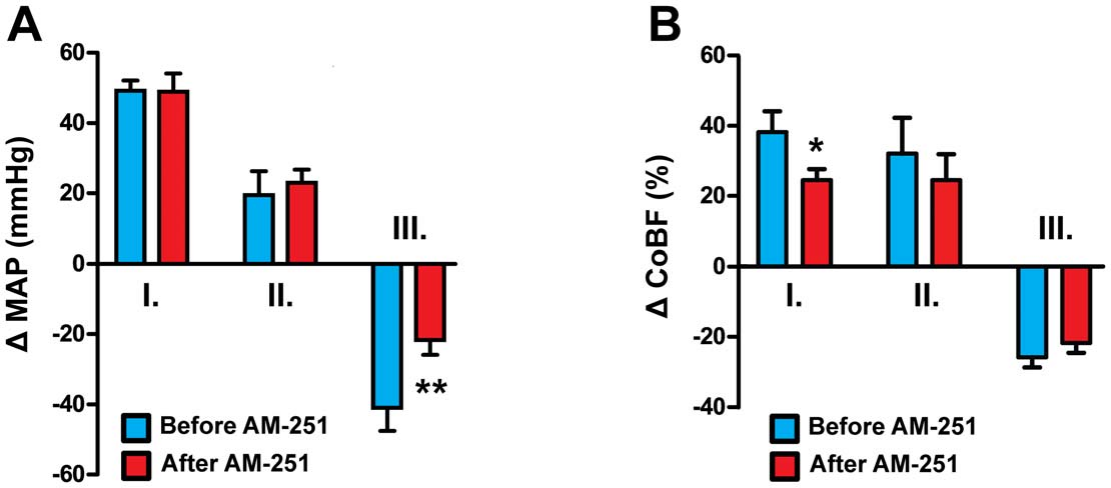
Effects of AM-404 on mean arterial pressure (A) and cerebrocortical blood flow (B) before and after AM-251 treatment. Changes in mean arterial pressure (MAP, n = 10) and cerebrocortical blood flow (CoBF, n = 20) are shown in phases I, II and III of the AM-404 (10 mg kg^−1^, i.v.) response before and after treatment with AM-251 (10 mg kg^−1^, i.v.). Values are means ± SEM and are expressed as changes from baseline (see Table 2); *P<0.05, **P<0.01, *versus* “Before AM-251”.

**Figure 4:**
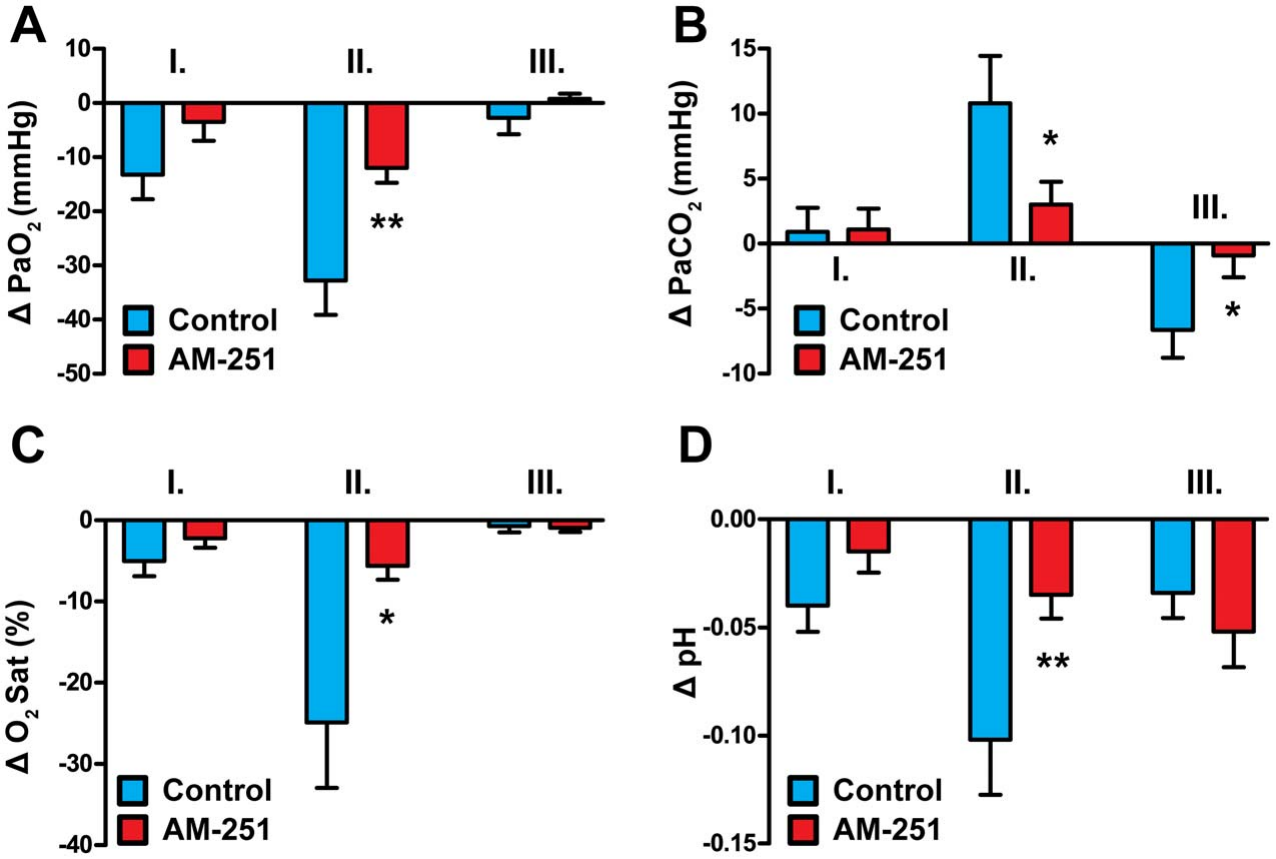
Effects of AM-404 on arterial blood gases and pH before and after AM-251 treatment. Arterial blood-gas and pH changes are shown in phases I, II and III of the AM-404 (10 mg kg^−1^, i.v.) response before and after treatment with AM-251 (10 mg kg^−1^, i.v.). Values are means ± SEM and are expressed as changes from baseline (see Table 2); *P<0.05, **P<0.01, *versus* “Before AM-251”, n = 10.

In the second part of the study we aimed to simulate the activation of the EC system by i.v. administration of the cannabinoid reuptake inhibitor AM-404 in a dose of 10 mg kg^−1^, which had been shown in previous studies to result in a more than 3-fold increase in the endogenous levels of anandamide in the mouse brain [38]. Baseline physiological parameters were within the normal range before AM-404 (Table 2). After inhibition of EC reuptake three different phases of the BP and CoBF changes could be detected (Figure 2). *Phase I* consisted of marked hypertension (Figures 2A and 3A) accompanied by a significant increase of CoBF (Figures 2B and 3B) with only minor changes in arterial blood gas tensions and pH (Figure 4). The BP and CoBF elevations reached their maximum within 0.5 min and thereafter started to return towards their baseline levels until the onset of the second phase with a delay of 1–2 min.

**Table 2:** Baseline physiological parameters before the administration of AM-404 in the absence and presence of AM-251.

	Before AM-251	After AM-251
**MAP (mmHg)**	112.8±6.5	103.8±3.0
**CoBF (AU)**	346.2±28.5	362.1±28.7
**PaO_2_ (mmHg)**	86.0±2.9	76.9±2.8*
**PaCO_2_ (mmHg)**	44.8±1.7	38.5±1.5*
**O_2_ Saturation (%)**	95.9±0.6	94.6±0.6*
**pH**	7.38±0.01	7.38±0.01

Values are mean ± SEM,

* P<0.05 *versus* “Before AM-251”, n = 10.

Among the changes of the cardiovascular parameters during *phase II* the elevation of CoBF was the most prominent (Figures 2B and 3B), accompanied by increased levels of expired CO_2_ (Figure 2C) and BP (Figures 2A and 3A). CoBF peaked 3.5 min after the administration of AM-404. Blood gas analysis revealed marked hypoxia (Figures 4A and 4C), hypercapnia (Figure 4B) and acidosis (Figure 4D) during this phase. Therefore, changes in the CoBF and BP were considered to be secondary to the hypoxia and hypercapnia due to the depression of respiration.

The *third phase* of changes induced by AM-404 was dominated by sustained hypotension (Figures 2A and 3A), which reached its maximum at 20 min; thereafter the BP started to return gradually towards its baseline levels. During this phase the arterial oxygen tension and saturation normalized (Figures 4A and 4C), whereas the previous hypercapnia was reverted to a slight hypocapnia (Figure 4B) and the acidic arterial pH returned towards the physiological level (Figure 4D). Interestingly, CoBF showed a significant decrease during phase III (Figure 3B), which was attributed to the above mentioned simultaneous reduction of the MAP and the arterial CO_2_-tension (PaCO_2_). BP and CoBF returned to their baseline levels within 45 min after the administration of AM-404.

In order to analyze the role of CB1-receptors in the mediation of effects induced by inhibition of EC reuptake, AM-251 was applied, and the administration of AM-404 as well as the subsequent measurements were repeated. In a control group of animals the effects of AM-404 were retested with the same protocol, except for replacement AM-251 by its vehicle. In these animals AM-404 induced identical effects during its two consecutive administrations (before and after the administration of vehicle) on the BP, CoBF, arterial blood gas tensions and pH (data not shown).

Baseline physiological parameters before administration of AM-404 in the presence of AM-251 were within the normal range, although O_2_- and CO_2_-tension were slightly lower as compared to the values at the beginning of the experiment (Table 2). In these animals all three phases of the AM-404 induced effects could be identified, but some of the changes (red bars in Figures 3 and 4) were significantly attenuated. During *phase I* the increase of MAP was identical before and after AM-251 (Figure 3A), indicating that CB1-receptors are not involved in the transient hypertension induced by enhanced EC levels. However, the increase of the CoBF attenuated significantly in the presence of AM-251 (Figure 3B). The minor changes in arterial blood gas tensions and pH during this phase were not altered significantly by AM-251 (Figure 4). Therefore, the smaller increase of CoBF in the presence of AM-251 in spite of the similar increase of BP may indicate an improved (or right-shifted) autoregulation of the cerebral circulation and that the higher CB1 activity caused by pharmacologically elevated EC levels after AM-404 sets the CoBF autoregulation at relatively lower BP values (see Discussion).

The most obvious effects of AM-251 were seen during *phase II*: both hypoxia and the hypercapnia as well as the acidosis were markedly attenuated (Figure 4), indicating that the enhanced levels of ECs after AM-404 suppresses respiration via CB1-receptor activation. In contrast, the mild hypertension during phase II was resistant to AM-251 (Figure 3A). Finally, the increase of CoBF was not different before and after AM-251 (Figure 3B), which is surprising if we consider that changes in blood gas and pH levels were suppressed by CB1-blockade. Therefore, we hypothesized that in addition to suppressing EC-induced hypoventilation, AM-251 enhanced the reactivity of the cerebrocortical circulation to H/H, a hypothesis that was tested in the last part of the study (see below).

In the *third phase* of AM-404 induced changes AM-251 significantly attenuated the hypotension (Figure 3A), indicating the involvement of CB1-receptors in mediating the effects of elevated EC levels. On the other hand, CB1-blockade failed to influence the decrease of CoBF during this phase (Figure 3B). The arterial O_2_ tension and saturation as well as the pH were also not influenced by AM-251, although the mild hypocapnia was attenuated (Figure 4).

### Effects of Endocannabinoids and CB1-receptors on the Increase in Cerebrocortical Blood Flow during Hypoxia and Hypercapnia

**Figure 5:**
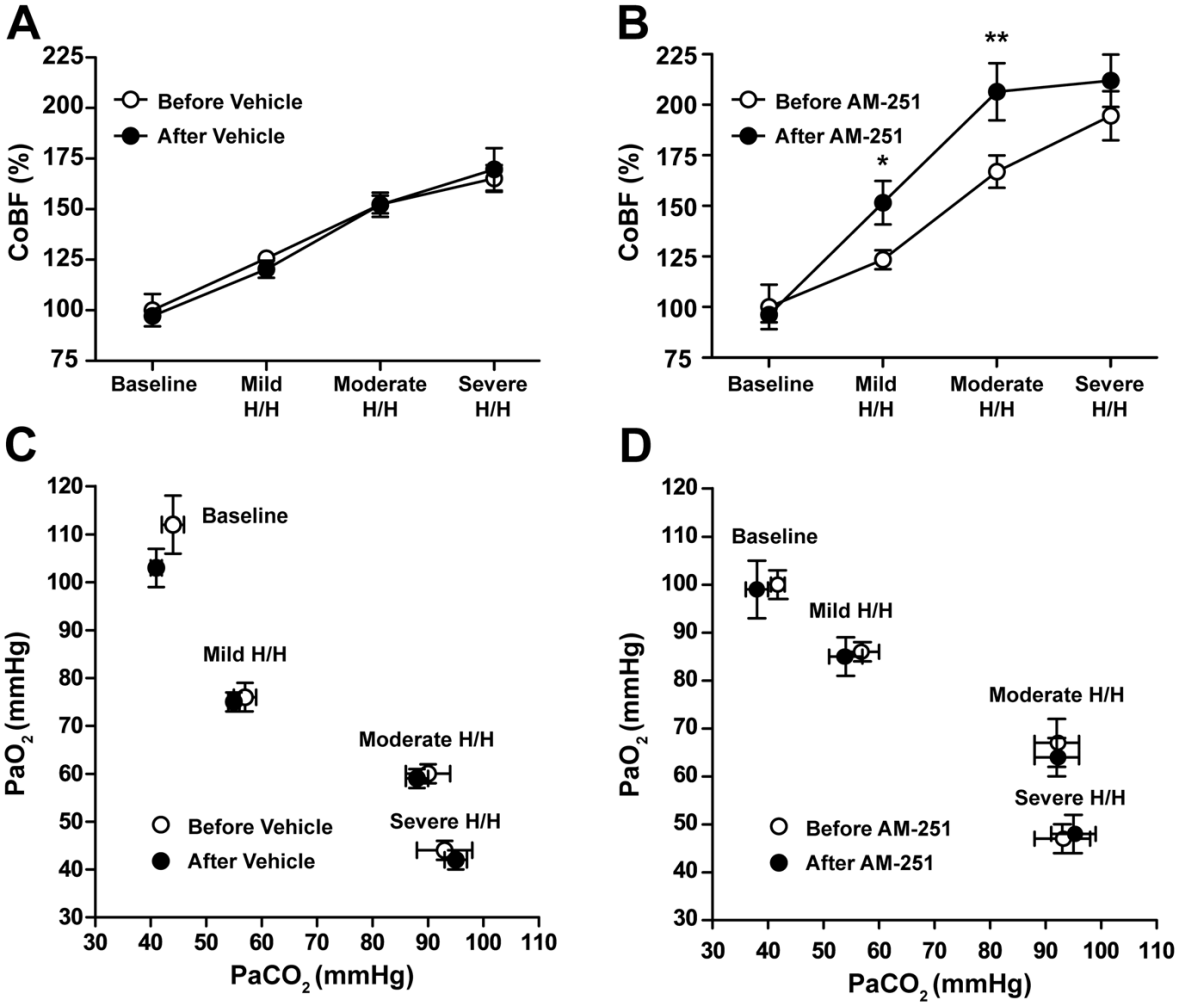
Cerebrocortical blood flow and arterial blood gas tensions during stepwise hypoxia/hypercapnia. Cerebrocortical blood flow (A and B, expressed as percentage of baseline levels) and arterial blood-gas tensions (C and D) are shown before (Baseline) and during mild, moderate and severe hypoxia/hypercapnia before (○) and after (•) intravenous injection of 10 mg kg^−1^ AM-251 or its vehicle. Values are mean ± SEM, *P<0.05, **P<0.01 *versus* “Before AM-251”, n = 4–12.

Since in phase II after AM-404 administration AM-251 failed to influence the cerebrocortical hyperemia in spite of the attenuation of H/H, we hypothesized that CB1-blockade may enhance the reactivity of CoBF to H/H. In order to test this hypothesis we produced stepwise H/H (as described in the Methods) before and after the administration of AM-251 or its vehicle, and determined the changes in CoBF. Inhalation of three different gas mixtures containing decreased O_2_- and increased CO_2_-content (as compared to air) induced reproducible levels of hypoxia and hypercapnia before and after the administration of AM-251 (Figure 5D) or its vehicle (Figure 5C), without significant changes in the BP (data not shown). The H/H-induced enhancement of CoBF was identical before and after the administration of the vehicle of AM-251 (Figure 5A). In contrast, AM-251 resulted in markedly increased CoBF changes during mild and moderate H/H (by 28.1±8.8% and 39.4±10.0%, respectively) without significantly influencing the peak CoBF during severe H/H (Figure 5B).

## Discussion

The cardiovascular actions of cannabinoids have been studied extensively since the identification of the biologically active constituents of marijuana (termed later as phytocannabinoids) in the 1960s, and the discovery of ECs in the early 1990s gave further impetus to the scientific endeavors in this field (for review see: [6], [39]–[41]). Cannabinoids are implicated in the control of systemic cardiovascular parameters such as blood pressure or cardiac output, as well as in the regulation of the regional vascular resistance and the blood supply to different organs and tissues including the cerebral circulation. However, literary data on the cerebrovascular effects of cannabinoids are controversial. Marijuana smoking has been reported to elevate CBF in correlation with the increasing plasma levels of Δ^9^-THC [42]. In accordance, Δ^9^-THC was able to increase the cerebral blood perfusion of dogs and humans [26]–[28], whereas both anandamide and the CB1 agonist HU-210 resulted in enhancement of the CBF in anesthetized rats, effects which could be prevented by the CB1 receptor antagonist SR141716A [32]. In contrast, conscious rats responded with a reduction of the CBF upon intravenous administration of Δ^9^-THC or anandamide [31], [43]. These discrepancies are not surprising, if we consider the variety of mechanisms by which cannabinoids may influence cerebral circulation. One obvious target is the cerebral vasculature itself, which appears to respond with vasodilation in a CB1-dependent manner [29], [30], [32]. On the other hand, cerebral circulation is tightly regulated by neuronal mechanisms [15], [16], [44], and in these pathways cannabinoids may act as modulators of the synaptic transmission. Finally, cannabinoids may have indirect effects on cerebral circulation by influencing the metabolic demand of neurons, or respiration and consequently blood gas tensions. These considerations also emphasize a major limitation of previous studies from the physiological point of view: the effects of exogenously applied phyto- or endocannabinoids may hardly resemble the functions of an endogenous control system. This revelation prompted us to use a different approach for elucidating the role of ECs in the regulation of the cerebral circulation by focusing on the changes elicited by suppressed or enhanced activity of the EC system. First we analyzed if the activity of the CB1-receptors has any tonic influence on the cerebrocortical circulation under resting conditions. For this purpose we applied a drug, AM-251, which acts both as an inverse agonist, thereby inhibiting the well-documented constitutive activity of CB1-receptors [45], [46], and also as an antagonist, thereby blocking the potential effects of resting EC release. In the second part of the study we aimed to simulate the activation of the EC system by administration of a cannabinoid reuptake inhibitor, AM-404. This approach has an unquestionable advantage over previous studies with systemic administration of phyto- or endocannabinoids, since after inhibition of reuptake the EC-levels increase in those tissue compartments which are physiologically exposed to these mediators. Nevertheless, as will be discussed below, our observations confirmed several cannabinoid-induced cardiovascular effects described in previous studies. Finally, in the third part of the study the effects of ECs and CB1-receptors on the H/H-induced changes of the CoBF were evaluated.

### Influence of Constitutive Endocannabinoid Release and CB1-receptor Activation on Systemic and Cerebrocortical Circulation

CB1-receptors have been reported to tonically modulate various physiological functions either by their constitutive activity or by mediating the effects of constitutively released ECs [45], [46]. In spite of the large number of studies addressing the role of cannabinoids in cardiovascular regulation [6], [39]–[41], the potential tonic influence of CB1-receptors on systemic or cerebral circulation has not been investigated yet. Therefore, the first aim of our study was to clarify this question by using AM-251, which works both as an antagonist and as an inverse agonist at CB1-receptors. In previous studies AM-251 was reported to inhibit basal G-protein-activity in rat cerebellar membranes [47], to enhance electrically evoked glutamate release from rat cerebellar neurons [48], and to suppress food intake and food-reinforced behavior in rats [49]. In the present study, however, we did not observe any significant effect of AM-251 on the systemic or cerebral circulation, in contrast to the pronounced effects seen after the activation of the EC system (see below). Therefore, it appears that CB1-receptors have no constitutive influence on the cardiovascular system under steady-state resting conditions, at least in healthy normotensive rats, which is consistent with reports on the normal hemodynamic profile of CB1-knockout mice [50], [51]. On the other hand, it is well known that both systemic BP and CBF are vital parameters of homeostasis, and therefore several backup regulatory mechanisms are involved in their maintenance. For this reason we cannot exclude the possibility that CB1-mediated pathways do contribute to steady-state BP- or CBF-regulation and that, when they are blocked pharmacologically or genetically, other control mechanisms take over their function.

### Influence of Enhanced Endocannabinoid Levels on the Systemic and Cerebrocortical Circulation

In the second part of the study enhanced activity of the EC system was simulated by administration of the EC reuptake inhibitor AM-404, and consequent changes of the systemic and cerebral circulatory and respiratory parameters were determined. Although it is generally accepted that, after their release to the extracellular space, ECs are rapidly cleared by cellular uptake followed by metabolism, the relative importance of the two processes is a question of debate [52], [53]. AM-404 was originally designed to target cellular uptake [54], but subsequently it has also been identified as a substrate of the fatty acid amide hydrolase (FAAH), the key enzyme of anandamide metabolism [55], and to activate transient receptor potential vanilloid type-1 (TRPV_1_) channels [56]. Whatever its exact target, AM-404 has been demonstrated to increase the endogenous levels of anandamide in the brain [38], an observation that justified its use in our present study. Upon administration of AM-404 three different phases of the systemic and cerebral circulatory responses could be identified, which are discussed below individually.

The *first phase* of changes elicited by AM-404 administration consisted of a transient hypertension resembling findings of previous studies on the cardiovascular effects of i.v. applied anandamide in anesthetized rats [34], [57]–[63] and mice [64], [65]. Our results not only confirm these findings, but also indicate that activation of the EC system may effectively elevate BP. However, CB1 blockade failed to influence the hypertensive effect of AM-404 in our experiments and also that of anandamide in previous studies [34], [57], [59], [60], [62], [65], indicating that it is not mediated by CB1 receptors. The elevation of the BP and total peripheral resistance in response to anandamide is reportedly absent in TRPV_1_-receptor deficient mice [64], an observation that, together with the well-documented activation of TRPV_1_ by anandamide [66], clearly explains the mechanisms of EC-induced hypertension, at least in mice. In rats, however, although the TRPV_1_ agonist capsaicin induced similar hypertension as anandamide or methanandamide, only the non-selective TRPV blocker ruthenium red but not the TRPV_1_ antagonist capsazepine was able to inhibit the pressor response to anandamide [34], [61]. These findings together with the observation that nifedipine is also able to suppress anandamide-induced hypertension [34] indicate that ECs may induce vasoconstriction via TRPV (but probably not TRPV_1_) mediated depolarization and opening of L-type Ca^2+^-channels in the vascular smooth muscle of rats. On the other hand, it was also demonstrated that the hypertensive effect of anandamide was preceded by a transient rise in the activity of rostral ventrolateral medulla neurons [58], and that NMDA-receptors and β_2_-adrenoreceptors located in the central nervous system (CNS) appear to be involved in the development of hypertension [34]. Therefore it is likely that both CNS and peripheral vascular pathways contribute to the mediation of EC-induced hypertension, and that species differences may exist in the mechanisms involved.

It is noteworthy that during the first phase of the AM-404-induced effects, CoBF increased markedly, indicating that the hypertension exceeded the upper limit of the cerebrovascular autoregulation. However, since the mean arterial BP elevated only slightly above (to 163.4±6.3 mmHg) the reported upper limit of autoregulation (ca. 150 mmHg), the corresponding increase of the CoBF (37.4±5.9%) appears to be relatively high and may indicate that the AM-404 induced enhancement of EC levels compromised autoregulation at high BP. In this respect the small but significant reduction of cerebrocortical hyperemia in the presence of AM-251, in spite of the similar BP-elevation, is an interesting finding, and may indicate an influence of CB1-receptors on the autoregulation of cerebral circulation, at least during the acute and transient hypertension caused by pharmacological elevation of EC levels. However, it has to be noted that in our study we did not determine the exact range of CoBF autoregulation under physiological conditions (i.e. without inhibition of EC reuptake), and therefore further studies focusing on this question will be required to verify the potential roles of ECs and CB1-receptors in the autoregulation of the cerebral circulation.

The primary effect of AM-404 during *phase II* was the depression of respiration leading to changes of the arterial blood gas tensions and pH as well as to the consequent increase of CoBF. Previous studies have already demonstrated that phytocannabinoids [67]–[73] and ECs [59], [74]–[77] may suppress respiration, but our observation is the first to indicate that endogenously released cannabinoids have a strong influence on respiratory control, at least during the activation of the EC system. Several lines of evidence indicate that the cannabinoid-induced respiratory depression may be mediated by CB1-receptors. It was shown that the CB1-antagonist AM-281 can inhibit the respiratory effects of i.v. applied anandamide in rats [77], which is consistent with our finding that AM-251 suppressed changes in arterial blood gas tensions and pH in response to AM-404. Furthermore, i.v. administration of the specific CB1-agonists WIN-55212-2 and CP-55940 induced similar respiratory depression as did Δ^9^-THC in rats, and the effect of WIN-55212-2 could be blocked by the CB1-antagonist SR-141716A [75]. Administration of WIN-55212-2 to the cisterna magna [76] or to the rostral ventrolateral medulla oblongata [74] of rats also suppressed respiration; these effects were sensitive to CB1-antagonists, indicating that central CB1-receptors negatively modulate respiratory control circuits.

In light of the changes of arterial blood gas tensions and pH in phase II after AM-404 administration, the marked increase of the CoBF is not surprising in our present study. However, although CB1-blockade suppressed the changes related to respiratory depression, it failed to influence the consequent changes of CoBF. In order to explain this discrepancy we hypothesized that in addition to inhibiting the respiratory effect of AM-404, AM-251 also enhanced the reactivity of the CoBF to H/H, and these two opposite effects neutralized each other. This hypothesis was tested in the last part of our study, and the results will be discussed below.

During *phase III* after AM-404 administration the most pronounced effect was the reduction of BP. This finding is consistent with numerous data in the literature indicating a sustained hypotensive effect of phyto- and endocannabinoids as well as synthetic cannabinoid analogs [6], [39]–[41]. Interestingly, both CB1 [32], [34], [57], [59]–[62], [64], [65], [78] and yet unidentified abnormal cannabidiol receptors [63] have been implicated in mediating this effect, whereas the involvement of CB2 and the putative abnormal cannabidiol receptor GPR55 has been excluded [79], [80]. Several lines of evidence indicate that CB1-mediated inhibition of noradrenaline release from postganglionic sympathetic neurons in the heart and vasculature is the main mechanism of cannabinoid-induced hypotension [58], [59], [62], [81]. Our finding that CB1-blockade inhibits the depressor effect of AM-404 supports this conclusion, but does not exclude the presence of a peripheral non-CB1-mediated action, since AM-251 failed to completely abolish the hypotension. Nevertheless, the fact that AM-404 resembled the effects of exogenously applied cannabinoids clearly indicates that activation of the EC system may decrease systemic vascular resistance and BP *in vivo*.

The reduction of CoBF during phase III prior to administration of AM-251 was likely to be induced by two simultaneous mechanisms. The first obvious mechanism is the reduction of the BP close to or slightly below the lower limit of autoregulation. The second mechanism could be a cerebrovascular contraction due to the reduction of the arterial CO_2_-tension, the most plausible explanation for which is that the H/H and acidosis during phase II activated the chemical regulation of respiration, at least in the absence of AM-251. If so, the attenuation of AM-404-induced hypocapnia in phase III by AM-251 could be due to the correspondent attenuation of AM-404-induced respiratory depression in phase II. With respect to CoBF, it is likely that the two indirect effects of AM-251 (i.e. prevention of hypocapnia and attenuation of hypotension) neutralized each other, resulting in an unaltered CoBF decrease in phase III. On the other hand, we cannot exclude the possibility that CB1-blockade impaired the autoregulation of the cerebrocortical circulation also at low BP levels. (As discussed above, in the presence of AM-404, AM-251 appeared to improve the autoregulation at high BP during phase I.) The involvement of CB1-receptors in the autoregulation of the cerebral circulation, at least during the activation of the EC system, would not be surprising, since several lines of evidence indicate a neuronal component in the cerebrovascular adaptation to changes in the systemic BP [15], [16], [82], but further studies will be required to test this potential interaction.

### Influence of CB1-receptors on the Cerebrocortical Circulation during Hypoxia and Hypercapnia

The last part of our study was devoted to investigating the role of CB1-receptors in H/H-induced cerebrocortical hyperemia. In spite of the fact that the enhancement of CBF during H/H was the first well-described reaction of the cerebral circulation, its mechanism is still poorly understood. Early researchers favored the idea of a negative feed-back system, in which decreased O_2_-tension or increased CO_2_-tension in the brain, as a consequence of insufficient CBF or enhanced metabolism of neurons, would initiate the release of vasodilator compounds (adenosine, H^+^, lactate, K^+^) in order to reset the balance between metabolic demand and energy supply. In the 1990s the role of endothelium-derived factors was proposed as a link between H/H and cerebrovascular smooth muscle relaxation. Recent advances of functional neuroimaging, however, called our attention to the pivotal role of neuronal mechanisms in the coupling of the CBF to the nutrient demand of the brain. Most notably it has been demonstrated in several studies that during neuronal activation the CBF changes precede the reduction of the O_2_-tension and the increase of the CO_2_-tension in the brain tissue, and a close interplay between neurons, astrocytes and microvessels (i.e. the “neurovascular unit”) is responsible for the effectiveness of this regulation [16], [83], [84]. Within this concept, increased glutamate release during enhanced synaptic activity would activate NMDA- and metabotropic glutamate receptors in postsynaptic neurons and neighboring astrocytes, and these cells would release arachidonic acid metabolites, NO and K^+^ leading to relaxation of the cerebrovascular smooth muscle [17], [84], [85].

In the present study we found that blockade of CB1-receptors enhances CoBF responses to H/H, indicating that ECs play an inhibitory role in this process. The involvement of cerebrovascular CB1-receptors in this effect can be excluded, since according to previous observations they mediate vasorelaxation in cerebral vessels [29], [30], [32]. On the other hand, it is well established that both neurons and astrocytes abundantly express CB1-receptors [86], [87], and therefore a CB1-mediated modulation of neuronal nitric oxid synthase (NOS) activity may explain our observations. It has been shown that CB1-agonists inhibit KCl-induced NOS-activation in cerebellar granule neurons without influencing the basal NO-release from these cells, and that the CB1-antagonist rimonabant both reversed the effect of CB1-activation and produced an increase in NOS activity that was additive with KCl [88]. Furthermore, CB1-receptors reportedly inhibit both glutamatergic transmission [86] and the metabolic activity of neurons and astrocytes [89], effects that may influence the release of NO and other vasoactive mediators and consequently alter CoBF. Astrocytes are potential oxygen-sensors of this control system, since it is well established that hypoxia suppresses glutamate uptake by astrocytes [90], which may result in activation of the glutamate receptor-mediated release of vasoactive mediators from neuronal elements of the neurovascular unit. Moreover, it is well documented that the cerebrovascular responses to H/H are modulated by sympathetic perivascular nerves [19], [91]–[94], a pathway that may also be influenced by the EC system and CB1-receptors [95], [96]. It is noteworthy that *in vivo* AM-404 treatment was shown to suppress *ex vivo* NO production in sciatic nerve homogenates, indicating that ECs may negatively regulate NO release from nitroxidergic nerves [97]. Whatever is the exact mechanism by which CB1-receptors modulate H/H-induced cerebrocortical hyperemia, our results appear to support the pivotal role of neuronal regulation of CoBF during H/H.

In conclusion, our study indicates that under resting physiological conditions CB1-receptor mediated mechanisms have limited tonic influence on the systemic and cerebral circulation. In agreement with previous studies investigating the systemic effects of intravenous administration of anandamide (reviewed in [4], [98]), we found that acute enhancement of EC levels by inhibition of cannabinoid cellular reuptake leads to transient increase of blood pressure followed by more sustained hypotension, as well as to suppression of the respiration; and that these responses, except for the initial hypertension, are mediated by CB1-receptors. Most importantly, our data suggest that the EC system and CB1-receptors play an important role in the regulation of the cerebral circulation during hypoxia and hypercapnia and may potentially be involved in the autoregulation of CBF.
